# Electrocardiogram classification using TSST-based spectrogram and ConViT

**DOI:** 10.3389/fcvm.2022.983543

**Published:** 2022-10-10

**Authors:** Pingping Bing, Yang Liu, Wei Liu, Jun Zhou, Lemei Zhu

**Affiliations:** ^1^Academician Workstation, Changsha Medical University, Changsha, China; ^2^College of Mechanical and Electrical Engineering, Beijing University of Chemical Technology, Beijing, China

**Keywords:** ECG classification, vision transformer, convolutional neural network, time-reassigned synchrosqueezing transform, class imbalance

## Abstract

As an important auxiliary tool of arrhythmia diagnosis, Electrocardiogram (ECG) is frequently utilized to detect a variety of cardiovascular diseases caused by arrhythmia, such as cardiac mechanical infarction. In the past few years, the classification of ECG has always been a challenging problem. This paper presents a novel deep learning model called convolutional vision transformer (ConViT), which combines vision transformer (ViT) with convolutional neural network (CNN), for ECG arrhythmia classification, in which the unique soft convolutional inductive bias of gated positional self-attention (GPSA) layers integrates the superiorities of attention mechanism and convolutional architecture. Moreover, the time-reassigned synchrosqueezing transform (TSST), a newly developed time-frequency analysis (TFA) method where the time-frequency coefficients are reassigned in the time direction, is employed to sharpen pulse traits for feature extraction. Aiming at the class imbalance phenomena in the traditional ECG database, the smote algorithm and focal loss (FL) are used for data augmentation and minority-class weighting, respectively. The experiment using MIT-BIH arrhythmia database indicates that the overall accuracy of the proposed model is as high as 99.5%. Furthermore, the specificity (Spe), F1-Score and positive Matthews Correlation Coefficient (MCC) of supra ventricular ectopic beat (S) and ventricular ectopic beat (V) are all more than 94%. These results demonstrate that the proposed method is superior to most of the existing methods.

## Introduction

Electrocardiogram (ECG) is a diagnosis and treatment technology to detect cardiac physiological activities by extracting human skin electrode signal. By analyzing ECG signal, doctors are able to correctly diagnose various arrhythmias, and then help to judge myocardial infarction, myocarditis, myocardial ischemia, pericardial effusion and other diseases. Therefore, exploring the internal characteristics of ECG is of great significance for the timely diagnosis and treatment of arrhythmia diseases (1, 2).

In the past decade, with the development of artificial intelligence, many machine learning methods mainly based on feature extraction and modal classification have achieved fruitful results in the application of ECG analysis. The works for ECG feature extraction include digital filtering (3), group optimization (4) and time-frequency analysis (5–8). Ozbay et al. combined the fuzzy C-means clustering algorithm (FCMA) and discrete wavelet transform to extract the key feature of ECG signal (9). Alickovic and Subasi used the multi-scale principal component analysis (PCA) to denoise ECG signal, and further extracted feature through autoregressive model (10). Azia et al. (11) applied empirical mode decomposition (EMD) and support vector machine (SVM) to region of interest extraction and signal denoising. In (12), the wavelet transform was utilized for data preprocessing, and then the PCA was added to project it to the lower dimensional feature space with particle swarm optimization. Marinho et al. (13) explored the combined advantages of different feature extraction methods and several classical machine learning models, and evaluated the actual achievements of Fourier transform, gerzel algorithm, higher order statistics and structural co-occurrence matrix on four types of perceptron: support vector machine, multi-layer perceptron, naive bayes model and optimum-path forest. Coast et al. (14) used the hidden Markov models to analyze cardiac arrhythmia. Osowski et al. (15) utilized the support vector machine to recognize heartbeat. Yeh et al. (16) developed a clustering method to identify ECG signal with arrhythmia. Park et al. (17) proposed the logistic regression to automatically classify the ECG interval characteristics. Li and Min (6) completed ECG classification by combining wavelet packet transform and random forests. In summary, the most commonly used machine learning methods include hidden Markov model (14), support vector machine (13, 15), clustering algorithm (16, 17), logistic regression (18), random forest (6, 19) and naive Bayes (13, 20, 21). However, the above-mentioned techniques have many limitations in practical application; for instance, they rely heavily on manual feature extraction and require a lot of time and expertise.

In recent years, due to the end-to-end learning convenience of deep learning technique, it has also made great progress in ECG classification. Kiranyaz et al. (22) introduced a 1-D convolution neural network (CNN) to deal with ECG arrhythmia classification task. Li et al. (23) presented the general regression neural network to extract correlation patterns from ECG signal. On the basis of CNN, Acharya et al. (24) added data augmentation and noise filtering technique to strengthen fitting ability of the model. Sellami and Hwang (25) paid more attention to the problem of class imbalance, and showed the solicitude for the classification of various samples in batch processing through batch-weight loss. Atal and Singh (26) developed the deep CNN, modified by rider optimization algorithm, to implement the automatic classification of ECG. In addition, some studies used the practice of machine learning for reference and combined TFA with deep learning model, which greatly improved the accuracy and robustness of the model. In order to make full use of spatial information of 2-D image, Huang et al. (7) transformed the time-domain ECG signal into time-frequency domain by STFT, and then fed the time-frequency map to the neural network as input feature. Wang et al. (27) employed continuous wavelet transform (CWT) to implement preprocessing and designed a CNN framework to achieve the automatic ECG classification from 2-D spectrum. To pursue a more readable TFR as input feature, Ozdemir et al. (28) proposed a new method for detecting and predicting seizure based on synchrosqueezing transform (SST) and CNN. Furthermore, the enhancement of TFA methods, such as STFT, CWT and Hilbert-Huang Transform (HHT), for hand gesture intelligent classification was discussed in (29). An important conclusion is that the time-frequency resolution of 2-D spectrum has a direct influence on the classification based on deep learning model. Nevertheless, these methods mentioned often simply transform the representation of ECG time-domain signal, and lack of deep excavation of its characteristics, so as to introduce a preprocessing technique in line with its attributes. Besides, the deep learning model such as deep CNN is subject to the problem of network degradation, in which the training sets are easy to be saturated due to the complexity of the deep model, and are limited by the hard inductive bias of pure convolution layers, resulting in insufficient data information mining. Finally, most of the existing studies on ECG classification do attach importance to the class imbalance in applied database, the number of normal heart rate sample is often hundreds of times that of abnormal, which will produce serious over fitting problem.

In this study, since the signal characteristics corresponding to arrhythmia are usually reflected in the pulse of ECG, a TFA technique called time-reassigned synchrosqueezing transform (TSST) which can highlight the characteristics of pulse signal that will be used to extract ECG information, which transforms ECG in the time domain into time-frequency domain with the high frequency resolution. Then, the two-dimensional signal is transformed into picture and input into the convolutional vision transformer (ConViT) for classification. Aiming at the class imbalance problem mentioned previously, the smote algorithm is adopted to synthesize some small sample data for soft balance, and the focal loss (FL) is performed to further make up for the defect of class imbalance. The contributions of this paper are expressed as follows: (1) the TSST is employed for ECG data preprocessing to make full use of pulse information; (2) the ConViT with convolutional architecture and self-attention mechanism is used for ECG classification; (3) the smote algorithm and FL are adopted to deal with the ECG class imbalance problem.

The rest of this paper is organized as follows. Section Theory describes the fundamental principle of TSST algorithm, ConViT framework and treatments of imbalance problem. In Section Experiment, the experimental results and discussions are shown. The conclusions are drawn in Section Discussion.

## Theory

### Method overview

**Figure 1:**
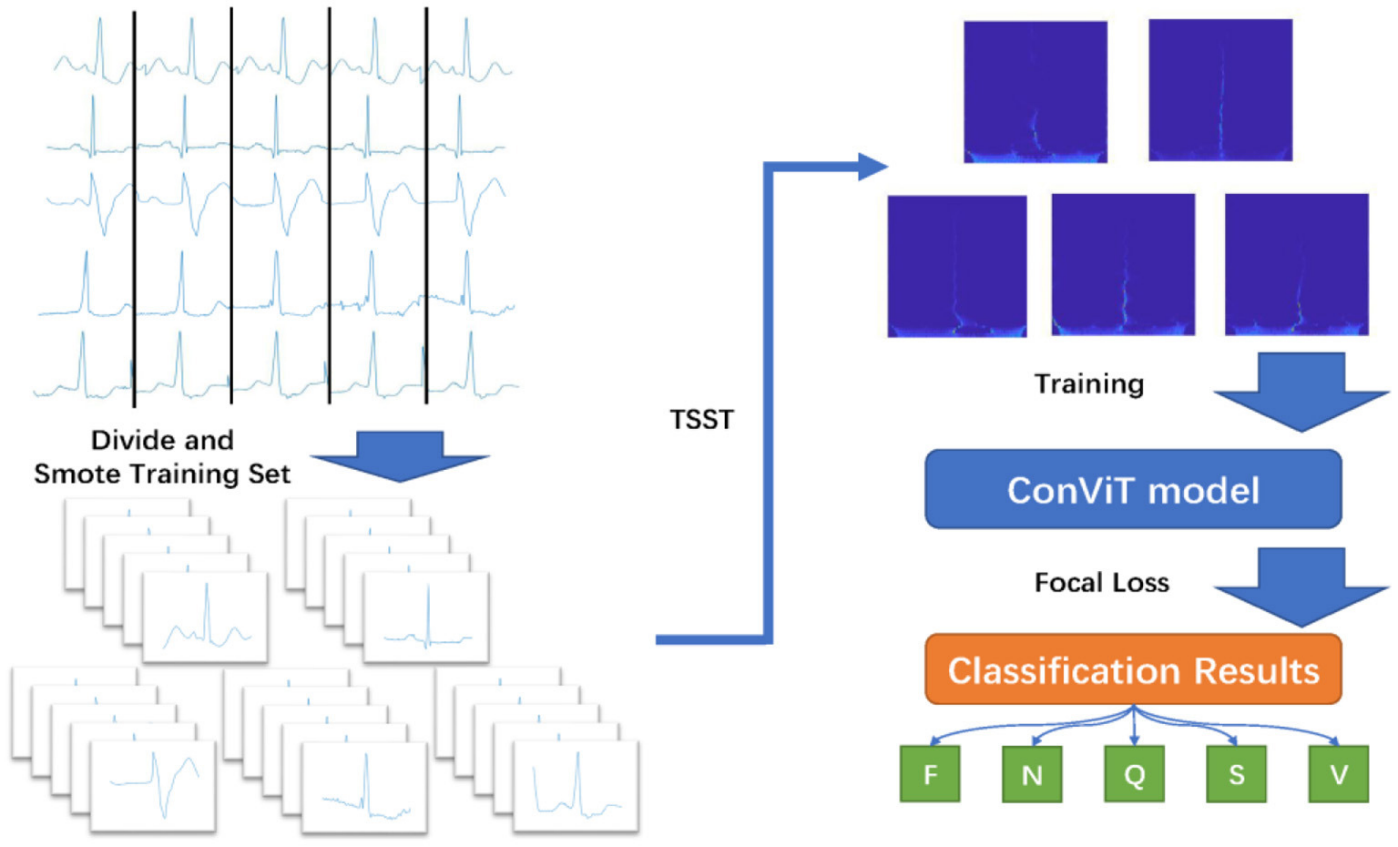
Flow chart of ECG classification based on TSST and ConViT.

The overall framework of the proposed ECG classification method in the paper is shown in Figure 1. The test data comes from MIT-BIH arrhythmia database (30). According to the R-wave position in the annotation file, a total of 300 points within the selected interval are taken as a time domain sample, and the data are enhanced by a small number of samples in the training set. Then, the TSST is utilized to transform the one-dimensional time-domain signal into two-dimensional time-frequency map, which will be input into ConViT with FL. Under the recommendations from Association for the Advancement of Medical Instrumentation (AAMI) (31), we will divide the original samples into five categories: fusion (F), non-ectopic beat (N), unknown (Q), supra ventricular ectopic beat (S) and ventricular ectopic beat (V), showing in Table 1, for the model processing.

**Table 1:** Details of MIT-BIH arrhythmia database.

**AAMI heartbeat class**	**MIT-BIH heartbeat type**	**MIT-BIH arrhythmia label**
F	fVN	F
N	N, LBBB, RBBB, AE, NE	N, L, R, e, j
Q	P, fPN, U	/, f, U
S	AP, aAP, NP, SP	A, a, J, S
V	PVC, VE	V, E

### Time reassigned synchrosqueezingtransform

TSST is a newly developed time-frequency decomposition algorithm (32). It reassigns the time-frequency coefficients along the time direction by calculating the group-delay estimator, so as to extract the transient characteristic of pulse signal, which is highly suitable for processing ECG signal. The definition and property of TSST are stated below.

The STFT of a signal*x*is defined as a function of time *t* and frequency ω computed with a Gaussian window*g*.


(1) $$F_{x}^{g}\left(t,\omega \right)=\int_{-\infty }^{+\infty }x\left(\tau \right)g^{*}\left(t-\tau \right)e^{-j\omega \tau }d\tau$$

where $g\left(t\right)=1/\sqrt{2\pi }e^{-t^{2}/2}$, and g^*^ denotes the complex conjugate of *g*. The time-frequency representation (TFR) corresponds to ${\left|F_{x}^{g}\left(t,\omega \right)\right|}^{2}$.

In order to further improve the resolution of TFR, a time reassignment step moves the energy of the signal according to the map $\left(t,\omega \right)\to \left({\hat{t}}_{x}\left(t,\omega \right),\omega \right)$, herein, ${\hat{t}}_{x}\left(t,\omega \right)$ is the group delay estimation mentioned above. The time reassignment operator $\hat{t}$ can be deduced as:


(2) $$\begin{array}{l} {\hat{t}}_{x}\left(t,\omega \right)=R\left(t-\frac{F_{x}^{\tau g}\left(t,\omega \right)}{F_{x}^{g}\left(t,\omega \right)}\right) \end{array}$$

where *R*(*Z*) stands for the real part of *Z*, τ*g*(*t*) = *tg*(*t*) is a modified version of the Gaussian window function *g*.

Therefore, TSST can be written as:


(3) $$S_{x}^{g}\left(t,\omega \right)=\int_{-\infty }^{+\infty }F_{x}^{g}\left(t,\omega \right)\delta \left(t-{\hat{t}}_{x}\left(t,\omega \right)\right)d\tau$$

**Figure 2:**
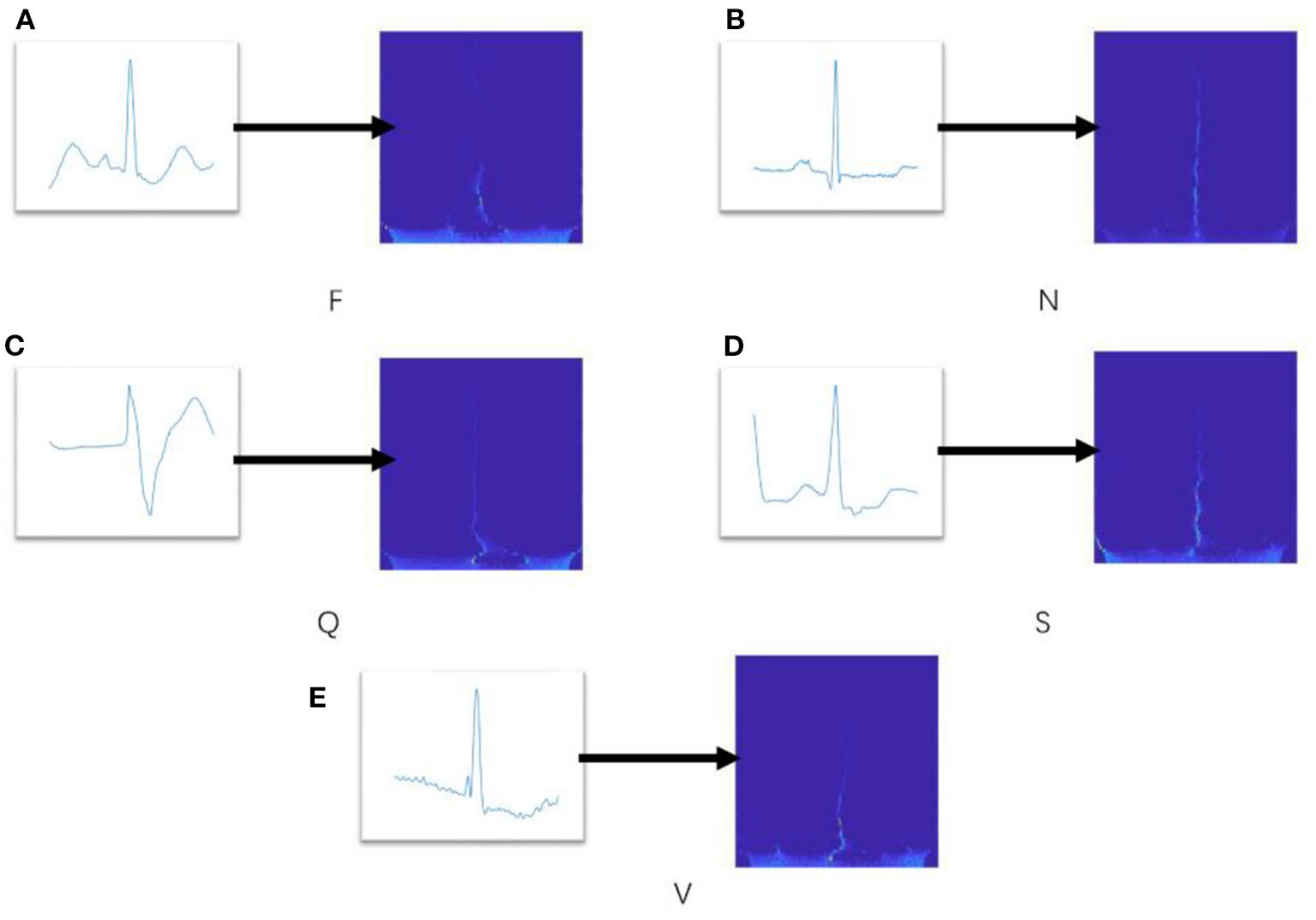
**(A–E)** Spectrograms of several ECG signals via TSST decomposition.

Next, the spectrogram ${\left|S_{x}^{g}\left(t,\omega \right)\right|}^{2}$ will be saved as picture and fed into the ConViT model as input sample. Figure 2 shows the spectrogram results, in which five representative time-domain ECG signals are transformed into two dimensional spectrograms through TSST. It can be seen that these spectrograms are characterized by high resolution in the time dimension, which is very beneficial for extracting the transient characteristics of ECG arrhythmia.

### Convit structure

**Figure 3:**
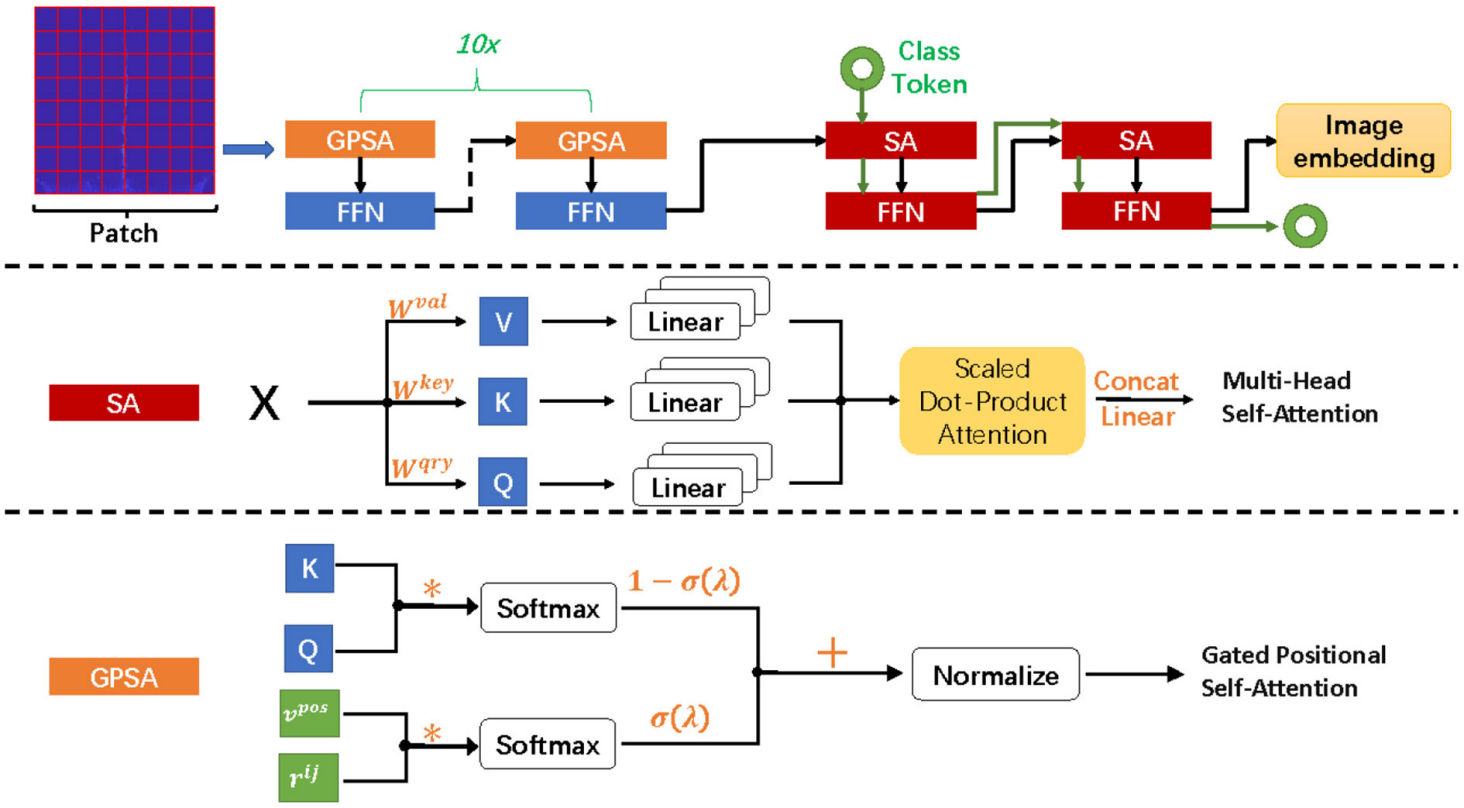
Framework of ConViT and the details of SA and GPSA.

ConViT combines the advantages of two popular neural network frameworks, CNN and Transformer (33–36), which overcomes the shortcomings of low performance upper limit caused by hard induction bias of CNN and the high dependence of Transformer on data. In the paper, the gated positional self-attention (GPSA) is employed to balance convolution and self-attention (SA) in a soft way, and its framework is shown in Figure 3. ConViT is based on vision transformer and consists of twelve propagation blocks composed of a SA layer and a two-layer feedforward network (FFN) with Gelu activation (see Figure 3). The difference is that the SA layer in the first ten blocks is replaced by GPSA layer, and the settings of SA layer are still retained in the last two blocks. In addition, the L2 regularization and dropout mechanism are applied in FNN to counter overfitting. Since the ECG spectrum is relatively simple, we set the input image with the size of 160 to 8 x 8 non-overlapping blocks of 20 x 20 pixels, and the embedding matrix dimension is 12.

For the SA layer, the essence of self-attention mechanism is to selectively manage the input through attention pooling. For single head self-attention with position, we can define it as *PSA*_*h*_, and *MHSA* performs concat and linear operations on *SA*_*h*_:


(4) $$\begin{array}{l} PSA_{ij}^{h}\left(K,Q,V\right):=V^{h}\text{softmax}\left(\frac{K_{i}^{hT}Q_{j}^{h}}{\sqrt{d}}+\upsilon_{pos}^{hT}r_{ij}\right) \end{array}$$

(5) $$\begin{array}{l} MHSA:{=}_{h\in \left[N_{h}\right]}^{concat}\left[SA_{h}\left(K,Q,V\right)\right]W^{out}+b^{out} \end{array}$$

where $\text{softmax}{\left(X\right)}_{ij}=\frac{e^{X_{ij}}}{\sum_{k}e^{X_{ik}}}$.

The input image is divided into multiple patches and represented as $X\in R^{D_{emb}\times N}$by embedding matrix processing. Therefore, we have *K* = *W*^*key*^*X*, *Q* = *W*^*qry*^*X*and *V* = *W*^*val*^*X*, here *W*^*key*^, *W*^*qry*^, *W*^*val*^ ∈ *R*^*D*×*Demb*^, *N*_*h*_ is the number of attention head. Trainable embedding $\upsilon_{pos}^{h}$ and relative position coding *r*_*ij*_ are added to discipline position information. Then, *D*_*emb*_ = *N*_*h*_*D*, $W^{out}\in R^{D_{emb}\times D_{emb}}$, $b^{out}\in R^{D_{emb}\times D}$. In (37), a PSA layer with *N*_*h*_ heads and a relative positional encoding of dimension *D*_*p*_ ≥ 3 can express any convolutional layer with filter size of $\sqrt{N_{h}}\times \sqrt{N_{h}}$.


(6) $$\left\{ \begin{array}{l} \upsilon_{pos}^{h}:=-\alpha^{h}\left(1,-2\Delta_{1}^{h},-2\Delta_{2}^{h}\right)\\ r_{\delta }={\left\|\delta \right\|}^{2},\delta_{1},\delta_{2}\\ W^{key},W^{qry}:=0,\;W^{val}=I \end{array}\right.$$

where α^*h*^ and $\Delta_{1}^{h}$, $\Delta_{2}^{h}$ determine the width and center of each attention head, respectively. (δ_1_, δ_2_) is a fixed value used to define the relative offset of *K* and *Q*.

Hence, each attention head only extracts local information to achieve the effect of convolution. However, this generalized convolution operation is difficult to be carried out on ViT, so GPSA is modified to allow it to decide whether to maintain convolution.


(7) $$\begin{array}{l} GPSA^{h}\left(K,Q,V\right):=V^{h}\text{normalize}\left[A^{h}\right] \end{array}$$

(8) $$\begin{array}{l} A_{ij}^{h}:=\left(1-\sigma \left(\lambda_{h}\right)\right)\text{softmax}\left(K_{i}^{hT}Q_{j}^{h}\right)\\ \;+\sigma \left(\lambda_{h}\right)\text{softmax}\left(\upsilon_{pos}^{hT}r_{ij}\right) \end{array}$$

where ${\left(\text{normalize}\left[{\text{A}}^{\text{h}}\right]\right)}_{ij}=\frac{A_{ij}}{\sum_{k}A_{ik}}$and $\sigma \left(Z\right)=\frac{1}{1+e^{-Z}}$.

The gating parameter λ is learned through the model, which is utilized to balance content-based self-attention and convolution initialization position self-attention, so as to achieve the effect of soft inductive bias.

### Treatment of class imbalance

In the actual situation, the amount of normal heart rate data is much larger than that of arrhythmia data. The problem caused by class imbalance is that the easy positive samples will make a major contribution to loss and dominate the update direction of the gradient. Hence, the model is unable to learn valid information for correct classification. In this paper, we introduce the smote algorithm and FL to combat it (38, 39). The former artificially generates a large number of scarce samples, and the latter pays attention to the samples that are difficult to be classified.

**Figure 4:**
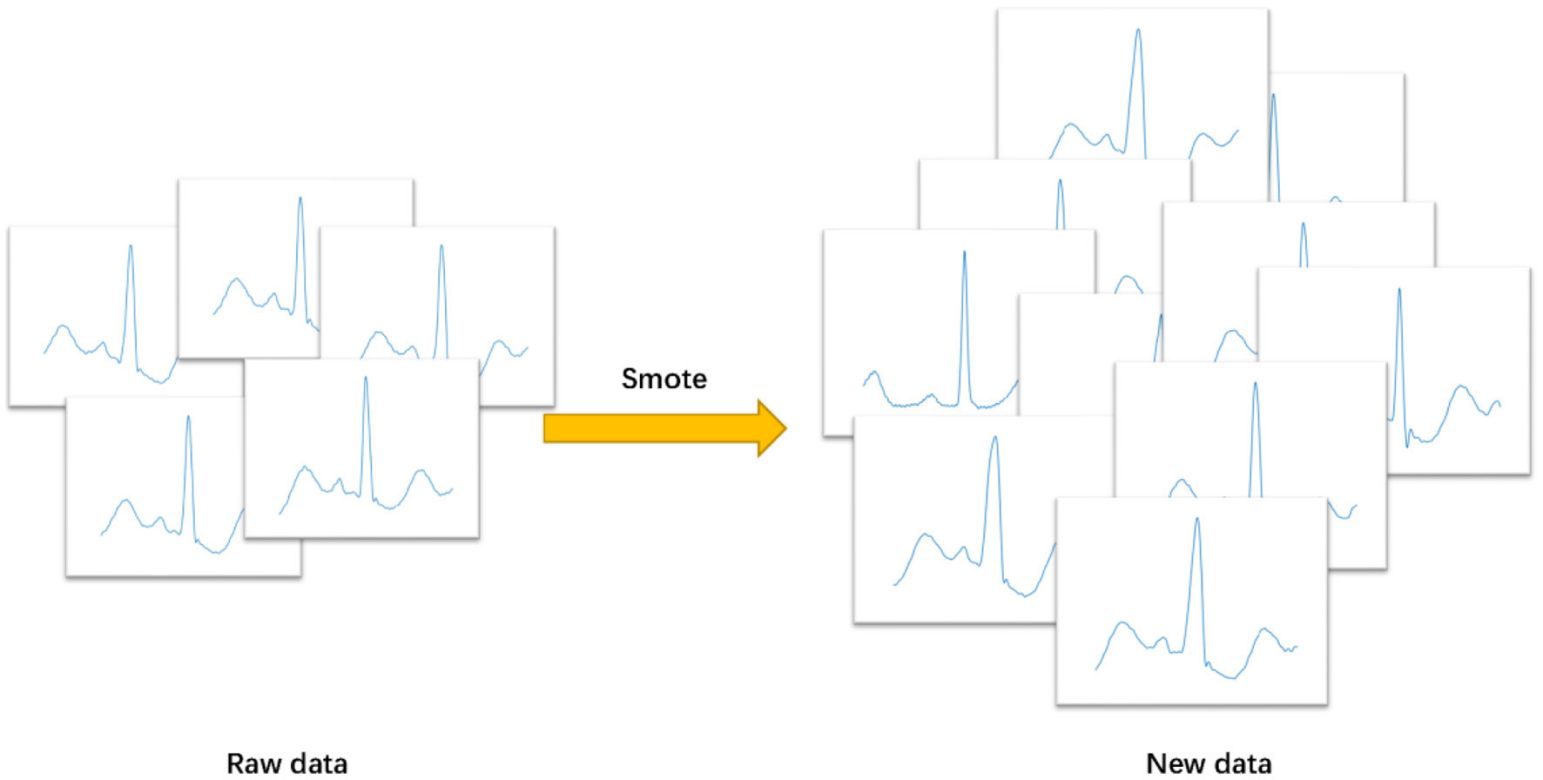
Smote result of class F samples.

Based on the *k* nearest neighbor points of each sample, smote algorithm randomly selects *N* adjacent points to multiply the difference by a threshold in the range of [0, 1], so as to achieve the purpose of synthesizing data. The core of this algorithm is that the feature of adjacent points in feature space is similar. It does not sample in the data space, but in the feature space, so its accuracy will be higher than the traditional sampling method. Figure 4 shows the data enhancement result of smote algorithm for class F samples. The formula for constructing new sample is as follows:


(9) $$\begin{array}{l} Z_{new}=Z+\text{rand}\left(0,1\right)*|Z-Z_{r}| \end{array}$$

where *Z* indicates the original sample, and *Z*_*r*_is the adjacent value randomly selected.

FL can be regarded as a loss function, which reduces the weight of samples easy to classify and increases the weight of samples difficult to classify. It focuses on training a sparse set of difficult samples. For multi-class classification task, FL can be defined as:


(10) $$\begin{array}{l} FL\left(p_{t}\right)=-\left(1-p_{t}\right)\log\left(p_{t}\right) \end{array}$$

(11) $$pt=\left\{ \begin{array}{l} \begin{array}{cc} x=p & y=1 \end{array}\\ \begin{array}{cc} y=1-p & y\neq 1 \end{array} \end{array}\right.$$

where *p*_*t*_ represents the probability predicted by the model as class *t*,*p* is the probability that the sample to be classified as positivity, and *y* is the output of the model. γ can adjust the rate of weight reduction of easy samples. The larger the γ, the more the loss of easy sample will be suppressed. It is worth noting that when γ = 0, FL is equal to the cross-entropy loss. In this example, γ = 2.

## Experiment

### Dataset description

In this paper, we employ MIT-BIH arrhythmia database to test the effectiveness of the proposed model, which includes 48 and a half hours of dual channel ambulatory ECG records of 47 subjects, with a sampling frequency of 360Hz and independent annotation by more than two experts.

**Figure 5:**
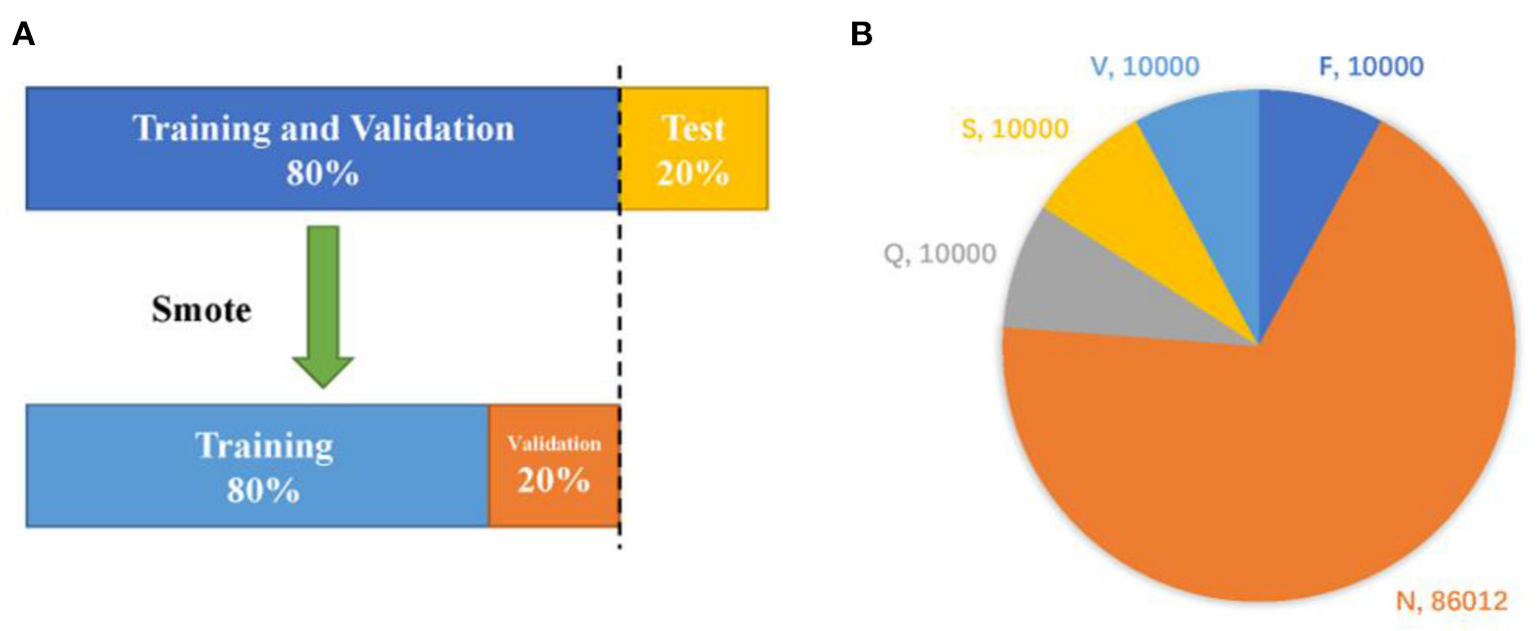
Dataset division strategy **(A)** and the quantity of samples before and after augmentation **(B)**.

In this example, we randomly divide the database into three parts. Firstly, the whole data is divided into training plus verification set and test set in the ratio of 8 to 2, then the former is augmented by smote algorithm and divided into training set and verification set in the same proportion. The data set division diagram and the number of samples (before and after data augmentation) (Table 1) are shown in Figure 5.

### Evalution

In order to further assess the validity of the proposed model in ECG classification task, the results of the test set are evaluated in terms of accuracy (Acc), sensitivity (Sen), specificity (Spe) Positive predictive value (Ppv), *F*1-Score and Matthews Correlation Coefficient (MCC), which are expressed as follows.


(12) $$\begin{array}{l} Acc=\frac{TP+TN}{TP+TN+FP+FN} \end{array}$$

(13) $$\begin{array}{l} Sen=\frac{TP}{TP+FN} \end{array}$$

(14) $$\begin{array}{l} Spe=\frac{TN}{TN+FP} \end{array}$$

(15) $$\begin{array}{l} Ppv=\frac{TP}{TP+FP} \end{array}$$

(16) $$\begin{array}{l} F1=\frac{Ppv\times Sen}{Ppv+Sen} \end{array}$$

(17) $$\begin{array}{l} MCC=\frac{TP\times TN-FP\times FN}{\sqrt{\left(TP+FP\right)\left(TP+FN\right)\left(TN+FP\right)\left(TN+FN\right)}} \end{array}$$

where TP, TN, FP and FN represent true positive, true negative, false positive and false negative, respectively.

## Result and discussion

**Figure 6:**
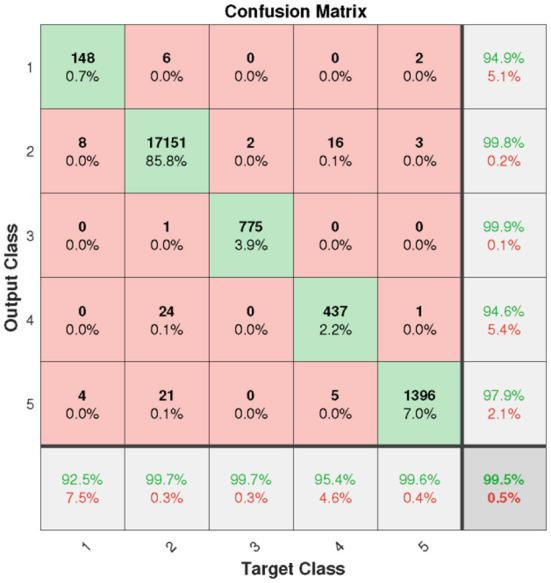
Confusion matrix of test set (1: F, 2: N, 3: Q, 4: S, 5: V).

In this section, the results will be discussed by means of confusion matrix, receiver operating characteristic curve (ROC), t-distributed stochastic neighbor embedding (t-SNE) and error histogram. Figure 6 shows the confusion matrix from the test set based on the proposed model. It can be clearly seen that the overall accuracy of our model is as high as 99.5%. However, due to the influence of FL on the weight of a small number of sample classes, the most class objects (class N) are probably incorrectly classified.

**Figure 7:**
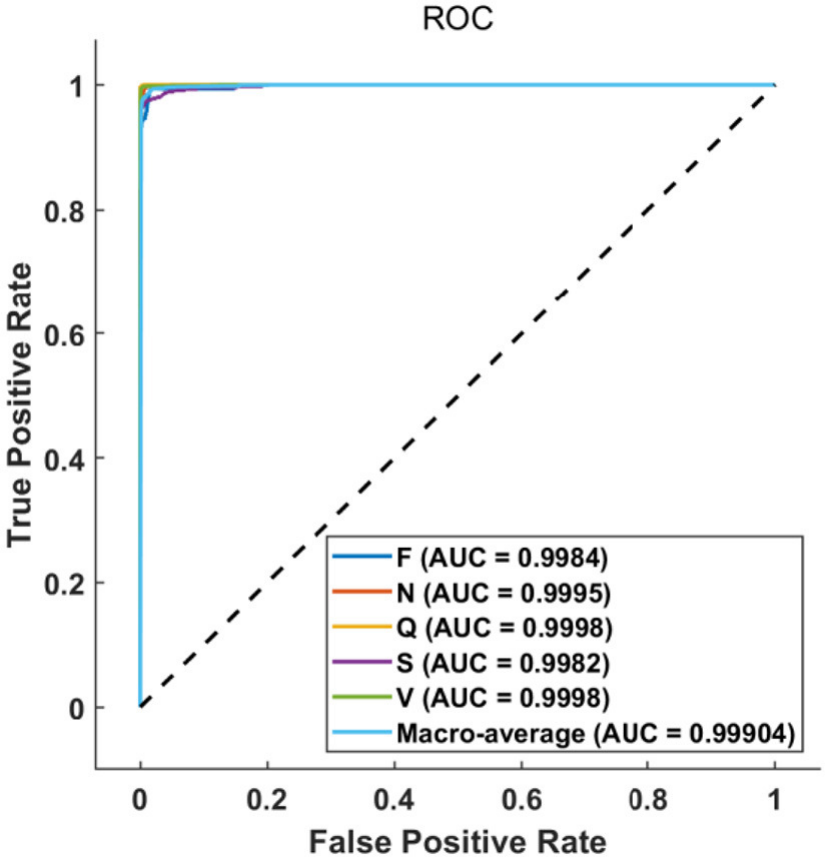
ROC of classification result and their AUCs.

The ROC curve in Figure 7 further illustrates the relationship between false positive rate (FPR) and true positive rate (TPR) of various classes. As can be observed, the performance of classes F and S is slightly poor owing to the small number of samples, the ROC curves of other classes are almost perfect. Nevertheless, all the area under curves (AUCs) are larger than 0.99, which indicates that the proposed method can achieve a satisfactory classification result.

**Figure 8:**
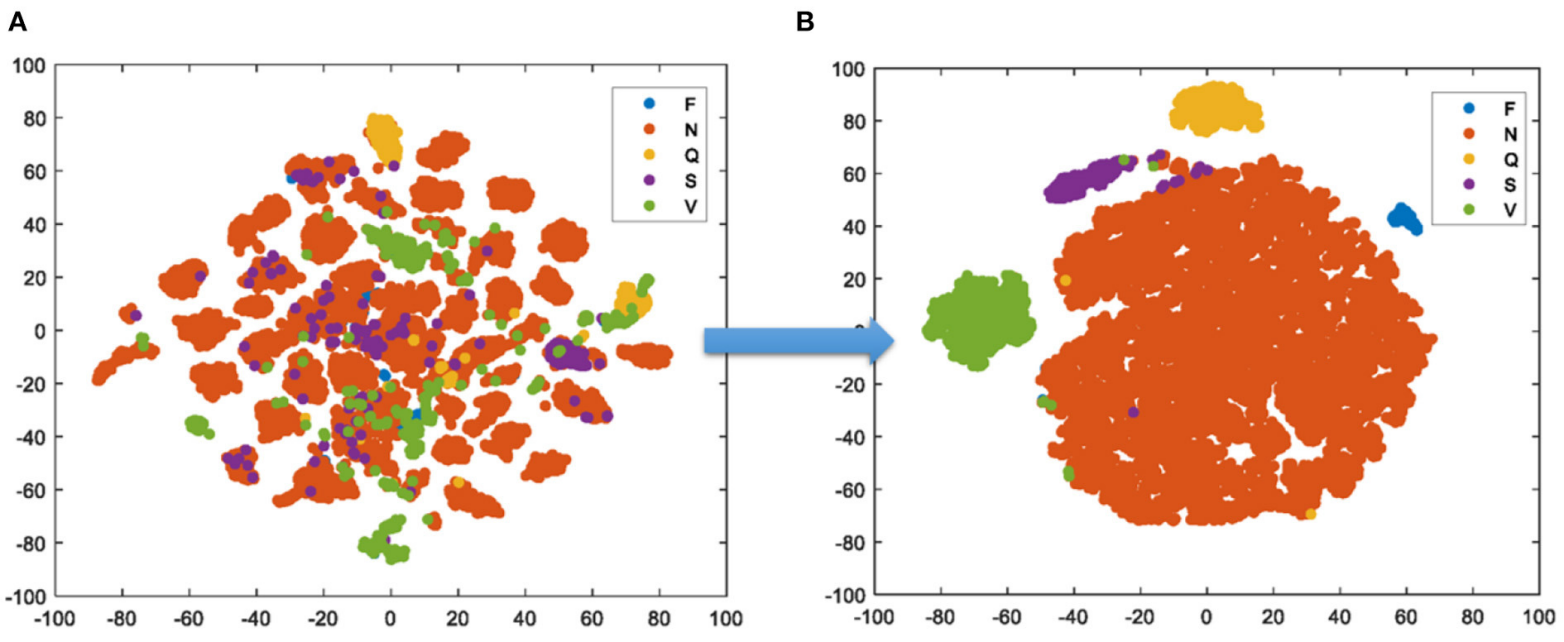
t-SNE results of input samples **(A)** and output samples **(B)**.

In Figure 8, the t-SNE gives the visualization result of the test set. It creates a compressed feature space, in which the similar samples are represented by the nearby points and the dissimilar samples are represented by far points with the high probability. Then, the Kullback Leibler divergence between the two distributions about the location of embedded points is minimized. Finally, the high-dimension data is simplified into a low-dimension graph with the affluent original information. One can clearly see that benefit from the feature extraction of TSST, the samples have been scattered well in space before the training, the proposed model achieves the excellent classification after the training.

**Figure 9:**
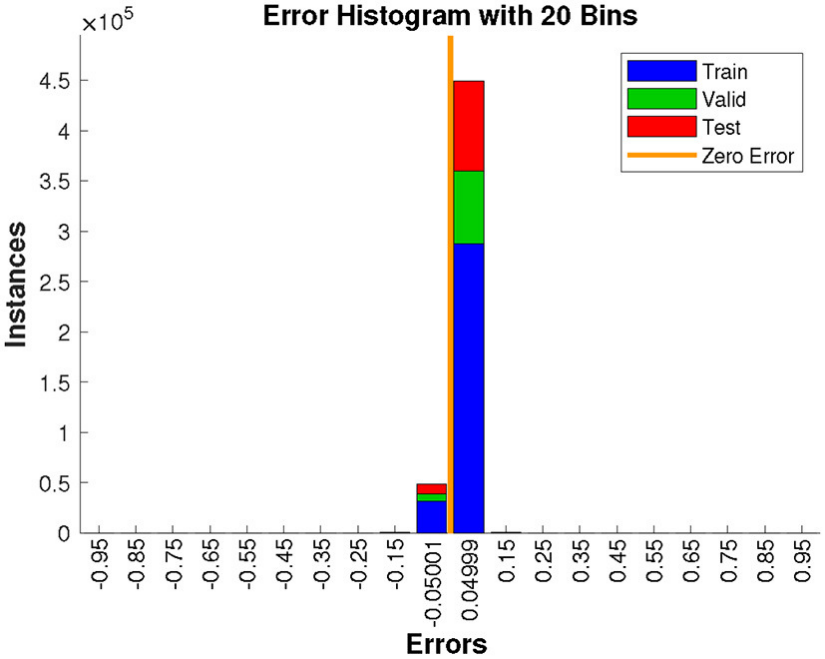
Error histogram (errors = output – target).

In addition, Figure 9 plots the error histogram, it shows that the proposed model has less prediction error, which further demonstrates the superior performance of the presented method.

**Figure 10:**
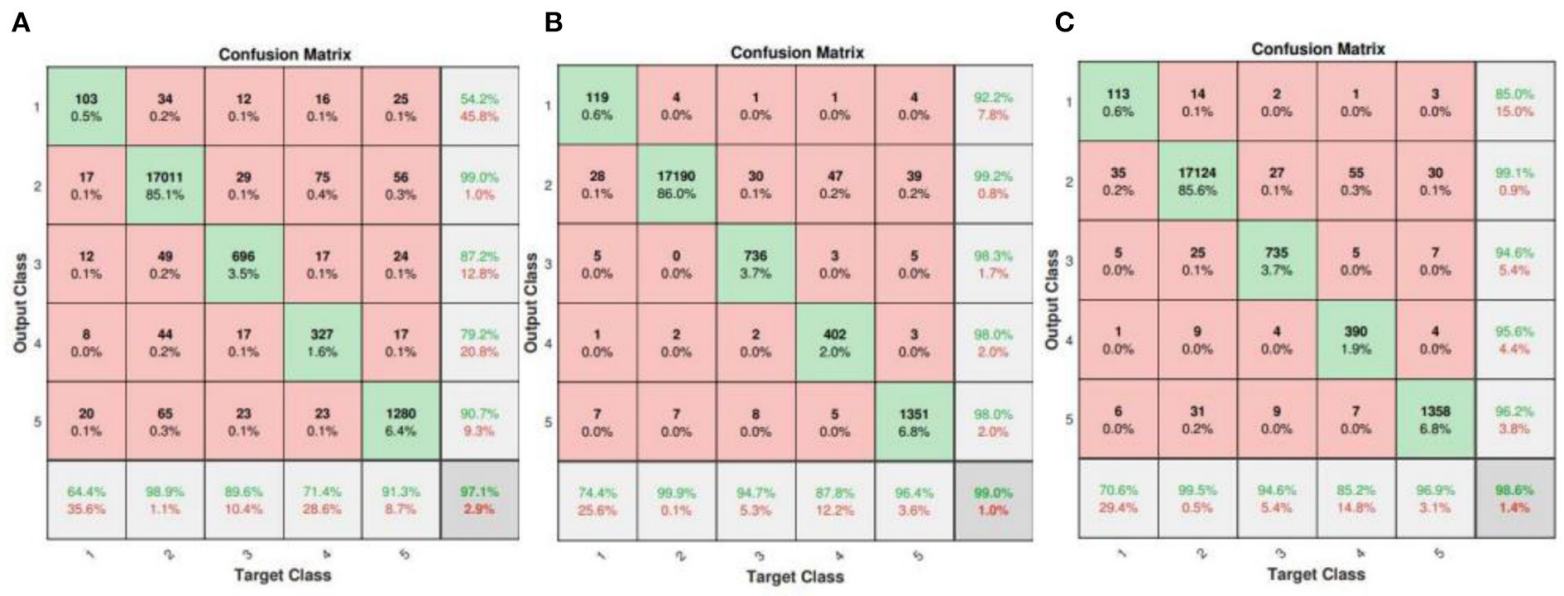
Confusion matrices of ConViT without TSST **(A)**, FL **(B)** and smote algorithm **(C)**, respectively.

On the other hand, the confusion matrix results of ConViT without TSST (each 1D ECG signal is simply stacked into 2D image), FL and smote algorithm respectively are given in Figure 10. It can be clearly seen that the overall performance of ConViT is far inferior to the scenario with TSST, which is likely due to the fact that the information from single time series is not enough to achieve the excellent classification. In addition, the scenarios without FL and smote algorithm, shown Figures 10A,C, indicate that the ConViT without balance processing generates a bias where the data is classified into N categories. Therefore, it is concluded that the classification result of few-shot without the above mentioned tricks is poor.

## Discussion

In this section, we apply our model to classification of classes S and V for comparison with other state-of-the-art methods in terms of Acc, Sen, Spe, F1-score and MCC, which is shown in Table 2. Note that the test set used in the paper consists of 20,000 beats of ECG. As illustrated in Table 2, the proposed method performs clearly better, with higher precision, which mainly benefits from the following three aspects: (1) TSST achieves the effective feature extraction on ECG signal; (2) FL and somte algorithm alleviate the conflict between the differences in various sample number; (3) Deep mining of input information by attention mechanism of ViT architecture and the CNN structure can ensure the property of small sample task. It should be mentioned that the proposed model implements 120 epochs on NVIDIA GeForce RTX 2060 about 9640s, which is suitable for a 2-D visual model with attention mechanism. Benefit from the ConViT, the model with multi-head attention mechanism can perform the fast iteration. Note that some important training parameters are listed in Table 3.

**Table 2:** Classification comparison of classes S and V.

**Approach**	***S***				***V***				**Data size**
	**Acc**	**Spe**	**F1-Score**	**MCC**	**Acc**	**Spe**	**F1-Score**	**MCC**	
CNN (22)	96.6	98.1	63.2	61.5	98.4	98.7	91.4	90.6	49,557
CNN Aug (24)	97.0	98.6	92.4	90.6	97.9	98.8	94.8	93.5	452,960
2-D CNN (40)	99.3	99.7	83.4	83.1	99.3	99.6	93.6	93.3	12,548
GRNN (23)	97.4	98.9	90.2	88.8	98.4	99.4	88.8	88.0	49,661
ResNet (41)	98.8	99.9	98.4	97.3	99.4	99.9	99.7	99.7	49,564
LSTM (27)	99.3	99.6	83.4	83.1	99.3	99.5	93.6	93.3	27,789
Proposed	99.7	99.9	95.0	94.9	99.7	99.7	97.7	97.5	20,000

**Table 3:** Training parameters.

**Learning rate**	**Batch size**	**Epoch**	**Embed dimension**	**Dropout rate**	**Decay rate**
1e-4	32	120	12	0.5	0.02/10epoch

To further verify the robustness of the proposed method, we apply the trained model with binary-classification (normal and others) to PTB database (47). The dataset contains 549 records of 290 subjects with 12 leads, which records the diseases including myocardial infarction (MI), cardiomyopathy/Heart failure, bundle branch block, dysrhythmia, myocardial hypertrophy, valvular heart disease, myocarditis, miscellaneous, healthy controls (normal). Each channel is sampled at the frequency of 1 kHz with 16-bit resolution. In this experiment, we apply ECG lead II data to TSST for test, which is focused on MI and healthy control data. The comparison results are listed in Table 4 Although not all indexes in the result of the proposed method are optimal, its overall performance is very competitive for an unseen dataset. The Acc of 94.6 is sufficient for MI diagnosis, which demonstrates the generalization of the proposed method again.

**Table 4:** Classification results of PTB database.

**Approach**	**Acc**	**Sen**	**Spe**	**Ppv**	**F1-Score**
KNN (42)	–	92.3	88.1	–	–
HMM with GMM (43)	82.5	85.7	79.8	–	–
ANN (44)	95.6	93.3	97.9	99.3	96.2
CNN (45)	93.5	93.7	92.8	98.0	95.8
ResNet (46)	92.6	93.2	92.0	–	–
Proposed	94.6	93.6	92.1	95.9	94.0

**Figure 11:**
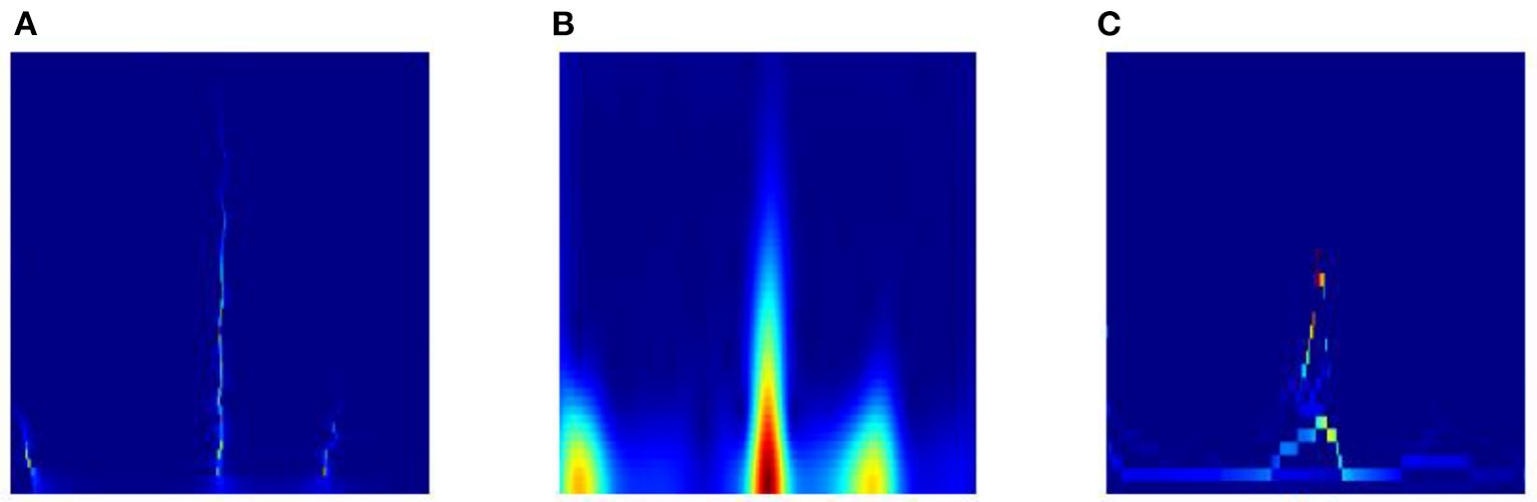
TFR of class S based on **(A)** TSST, **(B)** STFT and **(C)** EMD.

Third, we also list the results of class S based on TSST and traditional time-frequency analysis methods (e.g. STFT and EMD) in Figure 11. It is obvious that the TSST achieves a highly energy-concentrated TFR and highlights the pulse characteristics of ECG well compared with STFT, which helps to reduce some unnecessary convolution operations in the GPSA layer. Due to the existence of pulse points in ECG signal, EMD is easy to cause mode aliasing, as shown in the Figure 11(C), which is not conducive to feature extraction. In addition, the comparison results of TSST-, STFT- and EMD-based ConViT approaches for ECG classification using MIT-BIH dataset are shown in Table 5. The accuracy of ECG classification using TSST-based ConViT is 99.7%, which is obviously higher than STFT-based (95.6%) and EMD-based methods (92.1%). Similarly, the metrics, such as Spe, F1-Score and MCC, TSST-based ConViT also obtain the optimal values. The experiment indicates that TSST is a reliable technique for non-stationary signal, with pulse feature, processing and ECG classification in ConViT.

**Table 5:** Comparison results of TSST-, STFT- and EMD-based ConViT methods.

**TFA**	**S**				**V**			
	**Acc**	**Spe**	**F1-Score**	**MCC**	**Acc**	**Spe**	**F1-Score**	**MCC**
TSST	99.7	99.9	95.0	94.9	99.7	99.7	97.7	97.5
STFT	95.6	96.8	92.3	91.6	95.9	97.0	95.2	95.0
EMD	92.1	90.2	87.6	85.3	93.3	95.1	89.6	88.1

Actually, there are still some issues that need to be solved in the future. The first one is the adaptability of smote algorithm, traditionally used for 2-D image augmentation, for time series signals. Although the experiment (Figure 10) indicates that smote algorithm can improve ECG classification, the relevant research work is still lacking. The second one is about overfitting problem. We utilize some anti-overfitting strategies, such as L2 regularization and dropout, in the paper, but there are some differences in the classification performance for MIT-BIH and PTB datasets. Finally, more comparative experiments on the combination of TSST and deep learning models like (48) are needed, which can further illustrate the advantages of the proposed model, and this is also our future research direction.

## Conclusion

In this study, we propose a novel ECG classification method, it achieves the overall accuracy of 99.5% and does a better job classifying ECG signal compared to the traditional methods. With this method, the TSST transforms one-dimension ECG signal to two-dimension time-frequency map for characterizing the pulse characteristics of arrhythmia signal. The classifier performs smote algorithm and FL to deal with the class imbalance phenomenon. The former enhances the data by feature space sampling, and the latter ensures the classification ability by increasing the weight for a few class samples. In addition, as the main architecture of the model, on the one hand, ConViT utilizes multi-head attention mechanism of Transformer for image processing to make full use of the internal related information of the input. On the other hand, the hard induction bias of CNN enables the model to achieve good result with a few samples, and greatly improves the training speed.

## Data availability statement

The raw data supporting the conclusions of this article will be made available by the authors, without undue reservation.

## Ethics statement

Ethical review and approval was not required for this study in accordance with the local legislation and institutional requirements.

## Author contributions

PB: Conceptualization and software. LZ: Validation and formal analysis. JZ: Writing—review and editing and supervision. YL: Methodology and formal analysis. WL: Writing—original draft and writing—review and editing. All authors contributed to the article and approved the submitted version.

## Conflict of interest

The authors declare that the research was conducted in the absence of any commercial or financial relationships that could be construed as a potential conflict of interest.

## Publisher's note

All claims expressed in this article are solely those of the authors and do not necessarily represent those of their affiliated organizations, or those of the publisher, the editors and the reviewers. Any product that may be evaluated in this article, or claim that may be made by its manufacturer, is not guaranteed or endorsed by the publisher.
